# Correction: Imidazolium-based ionic liquids support biosimilar flavin electron transfer

**DOI:** 10.1039/d4ma90106a

**Published:** 2024-08-20

**Authors:** Grace I. Anderson, Alec Agee, Ariel L. Furst

**Affiliations:** a Department of Chemical Engineering, Massachusetts Institute of Technology Cambridge MA 02139 USA afurst@mit.edu; b Center for Environmental Health Sciences, Massachusetts Institute of Technology Cambridge MA 02139 USA

## Abstract

Correction for ‘Imidazolium-based ionic liquids support biosimilar flavin electron transfer’ by Grace I. Anderson *et al.*, *Mater. Adv.*, 2024, https://doi.org/10.1039/d4ma00558a.

The authors regret that an author’s name was incorrectly presented in the original manuscript. The corrected authorship list is as displayed above.

The Royal Society of Chemistry apologises for these errors and any consequent inconvenience to authors and readers.

